# Transcriptional characteristics and functional validation of three monocyte subsets during aging

**DOI:** 10.1186/s12979-023-00377-1

**Published:** 2023-09-27

**Authors:** Chen Wang, Yating Cheng, Boyu Li, Xueping Qiu, Hui Hu, Xiaokang Zhang, Zhibing Lu, Fang Zheng

**Affiliations:** 1 Center for Gene Diagnosis, Department of Laboratory Medicine, Zhongnan Hospital of Wuhan University, Wuhan, 430071 China; 2 Center of Clinical Laboratory, The First Affiliated Hospital of Soochow University, Suzhou, 215006 China; 3 Department of Cardiology, Zhongnan Hospital of Wuhan University, Wuhan, 430071 China

**Keywords:** Monocyte subsets, Cellular senescence, Senescence-associated secretory phenotype, Oxidative phosphorylation, Aerobic glycolysis

## Abstract

**Background:**

Age-associated changes in immunity are inextricably linked to chronic inflammation and age-related diseases, the impact of aging on monocyte subsets is poorly understood.

**Methods:**

Flow cytometry was applied to distinguish three monocyte subsets between 120 young and 103 aged individuals. We then analyzed the expression profiles of three monocyte subsets from 9 young and 9 older donors and CD14^+^ monocytes from 1202 individuals between 44 and 83 years old. Flow cytometry was used to measure β-galactosidase activities, ROS levels, mitochondrial contents, mitochondrial membrane potentials (MMPs) and intracellular IL-6 levels in three monocyte subsets of young and elderly individuals, and plasma IL-6 levels were detected by electrochemiluminescence immunoassay. Mitochondrial stress and glycolytic rate of CD14^+^ monocytes from young and aged individuals were measured by Seahorse XFe24 Analyzer.

**Results:**

Compared with young individuals, the percentage of classical subset in aged persons significantly decreased, while the proportion of nonclassical subset increased. Age-related differential genes were obviously enriched in cellular senescence, ROS, oxidative phosphorylation, mitochondrial respiratory chain, IL-6 and ribosome-related pathways. Compared with young individuals, the β-galactosidase activities, ROS contents, intracellular IL-6 levels of three monocyte subsets, and plasma IL-6 levels in aged individuals were significantly elevated, while the MMPs apparently declined with age and the mitochondrial contents were only increased in intermediate and nonclassical subsets. CD14^+^ monocytes from elderly adults had conspicuously lower basal and spare respiratory capacity and higher basal glycolysis than those from young individuals.

**Conclusions:**

During aging, monocytes exhibited senescence-associated secretory phenotype, mitochondrial dysfunction, decreased oxidative phosphorylation and increased glycolysis and the nonclassical subset displayed the clearest features of aging. Our study comprehensively investigated age-related transcriptional alterations of three monocyte subsets and identified the pivotal pathways of monocyte senescence, which may have significant implications for tactics to alleviate age-related conditions.

**Supplementary Information:**

The online version contains supplementary material available at 10.1186/s12979-023-00377-1.

## Background

Currently, the world population is encountering a rapid growth of elderly people. It is estimated that by 2050, there will be up to 400 million Chinese citizens aged 65 + , among them 150 million will be 80 + [1]. Age-induced deterioration and dysregulation of the immune system, also known as immunosenescence, has been associated with weakened responses to vaccines and increased frequency of infections, cancer, and cardiovascular and neurodegenerative diseases that lead to higher morbidity and mortality in older adults [2, 3]. In addition, immunosenescence can also be correlated with a state of low-grade, persistent and sterile chronic inflammation, referred to as inflammaging, which is manifested by elevated levels of pro-inflammatory cytokines, ultimately resulting in collateral damage to tissues and organs [3, 4].

The dysfunctions of immune system during aging present alterations in the abundance and function of immune cells implicated in the interaction between the innate and adaptive immunity. Among the innate cells, circulating monocytes, originated from the bone marrow myeloid precursors, are the most abundant, accounting for approximately 10% of all peripheral blood leukocytes in human [5]. Monocytes participate in the innate immunity against various pathogens via multiple pattern recognition receptors such as NOD1, MDA5, TLRs and RIG-I-like receptor [6, 7]. They activate and regulate the adaptive immune responses by phagocytosis, cytokine production, antigen presentation and differentiation into macrophages and dendritic cells [8–10]. Cellular senescence is a status of irreversible proliferative arrest in response to various stresses [11]. Although monocytes are generally considered to be non-proliferative owing to a short lifespan of about 3 days [12], transcriptomic profiling have indicated that classical monocytes are proliferative and anti-apoptotic in comparison with the intermediate and non-classical subsets [13]. In addition, the existence of a “proliferative monocyte” population was observed in vitro and this subpopulation was identified as CD14^+^ monocytes [14]. Intriguingly, a deuterium labeling study has implied that human monocytes can circulate in the blood stream for as long as 12 days [15]. Therefore, it is reasonable that monocytes may undergo cellular senescence during this period. Aging affects the cytokine secretion profiles of monocytes following different TLR ligands stimulation. Specifically, monocytes from older adults exhibit a weaker IL-1β and IFN-β response to LPS and influenza A virus treatment, respectively [16, 17]. Furthermore, LPS stimulation contributed to a reduced release of IL-1β and IFN-γ, while 5’pppRNA treatment triggered a decreased secretion of CCL8 and IFN-α in monocytes isolated from aged donors [18]. Additionally, poor response to cytokines is a common feature of monocytes and other immune cells in elderly individuals [19].

Monocytes consist of a heterogeneous population, which can be divided into three distinct monocyte subsets based on their expression of the surface markers CD14 and CD16: classical monocytes (CD14^+^CD16^−^), intermediate monocytes (CD14^+^CD16^+^) and nonclassical monocytes (CD14^−^CD16^+^), whilst mass cytometry and single-cell transcriptomic techniques have allowed for deeper subtype analysis of monocytes [20–22]. Classical subset takes a crucial role in the initiation and development of the inflammatory response, producing high levels of CCL2, IL-6, IL-8, IL-10 and reactive oxygen species (ROS) in response to pathogens [23]. In contrast, nonclassical subset seems to exert the anti-inflammatory functions, patrolling the endothelium of blood vessels and producing IL-1β, CCL3 and TNF-α in response to viruses and immune complexes [24]. Despite being considered as a transient differentiation stage between classical and nonclassical subsets, the intermediate monocytes are identified as main producers of proinflammatory cytokines (IL-1β and TNF-α) and ROS in response to lipopolysaccharide (LPS) and as highly antigen-presenting and proangiogenic cells [13, 23]. Moreover, three monocyte subsets exhibit distinct cytokine secretion profiles after stimulation with TLR ligands, further highlighting their discrete functions [18]. Consequently, the functional roles of these subsets and how these characteristics change with age are required to be fully elucidated.

## Results

### Hematological characteristics and monocyte immunophenotyping of study population

**Fig. 1:**
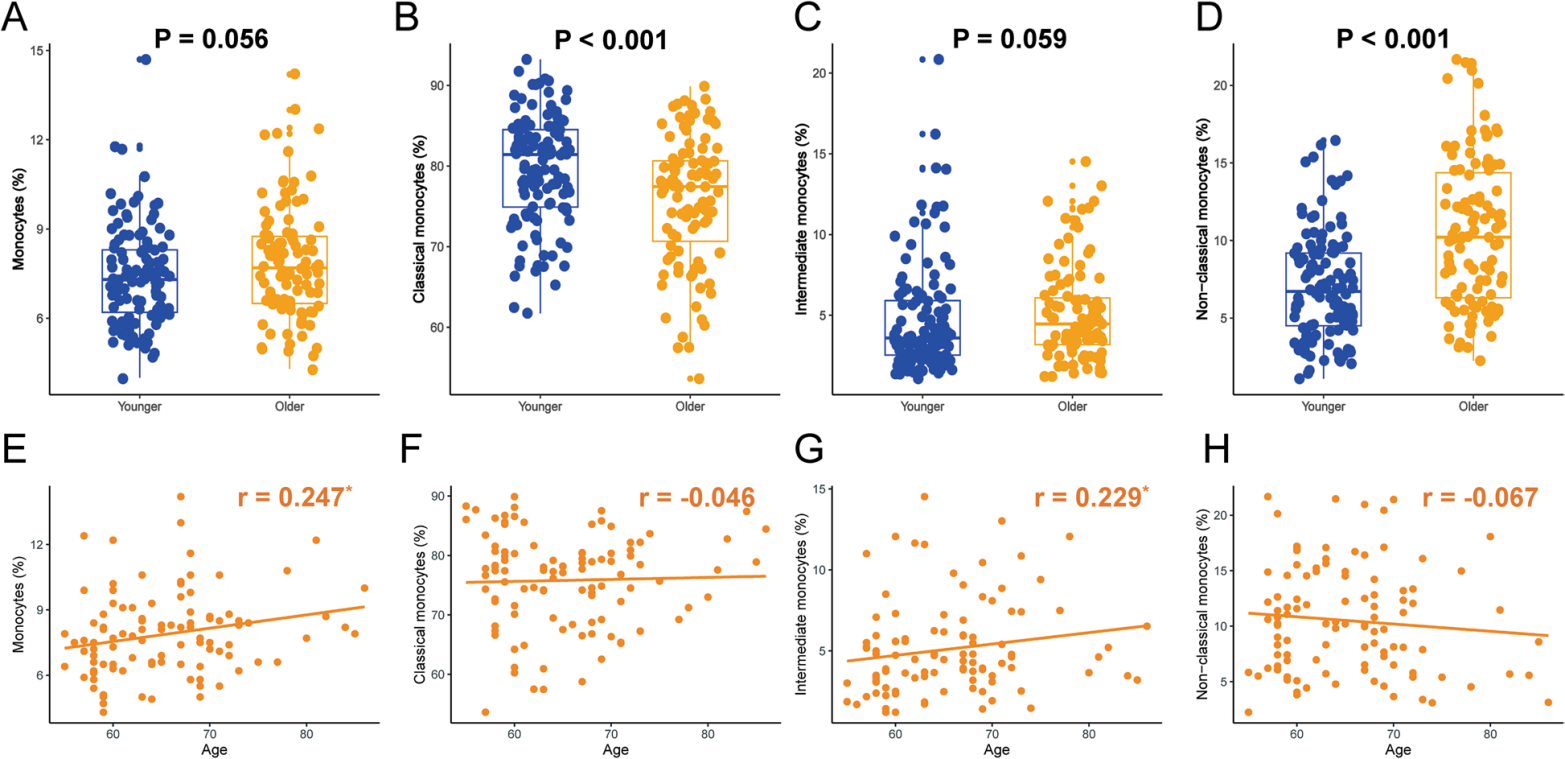
Comparisons of the proportions of monocytes and their three subsets in young (19–30 years, *n* = 120) and older (55–86 years, *n* = 103) individuals. **A**. Comparison of the percentage of monocytes between young and aged individuals. **B**. Comparison of the proportion of classical subset in young and elderly individuals. **C**. Comparison of the percentage of intermediate subset between young and aged individuals. **D**. Comparison of the proportion of nonclassical subset in young and older individuals. **E**. Correlation of the percentage of monocytes and age in the older group. **F**. Association of the proportion of classical subset and age in the elderly group. **G**. Correlation of the percentage of intermediate subset and age in the older group. **H**. Association of the proportion of nonclassical subset and age in the elderly group. Blue dots symbolize young individuals and orange dots denote aged individuals, ^*^P < 0.05

Complete blood counts and differential analysis of whole blood collected from healthy young (19–30 years old, *n* = 120) and older (55–86 years old, *n* = 103) donors indicated obvious decreases of RBC (4.78 ± 0.42 *vs* 4.53 ± 0.44, *P* < 0.001) and PLT counts (232.46 ± 55.41 *vs* 204.32 ± 50.58, *P* < 0.001) and notable increases of MCV (89.74 ± 4.04 *vs* 92.86 ± 4.06, *P* < 0.001), MCH (29.93 ± 1.61 *vs* 30.96 ± 1.57, *P* < 0.001), RDW (12.98 ± 1.15 *vs* 13.52 ± 0.74, *P* < 0.001) and BASO% (0.57 ± 0.31 *vs* 0.67 ± 0.31, *P* = 0.009) with age (Table 1). Intriguingly, total WBC count had no difference between the young and aged groups, but there were marginally significant alterations in WBC differentials: the percentage of total lymphocytes (35.94 ± 7.48 *vs* 33.98 ± 8.63, *P* = 0.07) declined with age (Table 1), while the proportion of total monocytes (7.40 ± 1.64 *vs* 7.87 ± 1.90, *P* = 0.056) was elevated (Fig. 1A), consistent with the previously depicted age-associated shift towards the myeloid lineage differentiation.

**Table 1:** Comparisons of hematological characteristics between young and aged groups

Indicators	Young cohort (*n* = 120)	Aged cohort (*n* = 103)	*P*
**Age (years)**	**24.30 ± 1.96**	**65.33 ± 6.99**	** < 0.001**
Gender, Male (%)	60 (50%)	53 (51.5%)	0.828
**RBC (× 10**^**12**^**/L)**	**4.78 ± 0.42**	**4.53 ± 0.44**	** < 0.001**
HCT (%)	42.81 ± 3.50	42.02 ± 3.49	0.154
**MCV (fl)**	**89.74 ± 4.06**	**92.86 ± 4.06**	** < 0.001**
**RDW (%)**	**12.98 ± 1.15**	**13.52 ± 0.74**	** < 0.001**
HGB (g/L)	142.78 ± 12.69	140.07 ± 12.09	0.134
**MCH (pg)**	**29.93 ± 1.61**	**30.96 ± 1.57**	** < 0.001**
MCHC (g/L)	330.92 ± 28.09	333.33 ± 6.40	0.998
WBC (× 10^9^/L)	6.02 ± 1.27	5.94 ± 1.64	0.232
NEUT (× 10^9^/L)	3.28 ± 1.10	3.28 ± 1.09	0.891
NEUT %	53.76 ± 8.12	54.95 ± 8.79	0.297
LYMPH (× 10^9^/L)	2.12 ± 0.51	2.01 ± 0.75	0.218
**LYMPH %**	**35.94 ± 7.48**	**33.98 ± 8.63**	**0.07**
MONO (× 10^9^/L)	0.44 ± 0.10	0.46 ± 0.15	0.69
**MONO %**	**7.40 ± 1.64**	**7.87 ± 1.90**	**0.056**
EO (× 10^9^/L)	0.14 ± 0.10	0.22 ± 0.68	0.282
EO %	2.34 ± 1.58	2.54 ± 1.62	0.276
BASO (× 10^9^/L)	0.02 ± 0.03	0.03 ± 0.03	0.110
**BASO %**	**0.57 ± 0.31**	**0.67 ± 0.31**	**0.009**
**PLT (× 10**^**9**^**/L)**	**232.46 ± 55.41**	**204.32 ± 50.58**	** < 0.001**
MPV (fl)	8.81 ± 1.00	8.98 ± 1.30	0.603

*Abbreviations*: *RBC* red blood cell count, *HCT* hematocrit, *MCV* mean corpuscular volume, *RDW-CV* red blood cell distribution width coefficient variation, *HGB* hemoglobin, *MCH* mean corpuscular hemoglobin, *MCHC* mean corpuscular hemoglobin concentration, *WBC* white blood cell count, *NEUT* neutrophil count, *NEUT%* percentage of neutrophils, *LYMPH* lymphocyte count, *LYMPH%* percentage of lymphocytes, *MONO* monocyte count, *MONO%* percentage of monocytes, *EO* eosinophil count, *EO%* percentage of eosinophils, *BASO* basophil count, *BASO%* percentage of basophils, *PLT* platelet count, *MPV* mean platelet volume

To further explore the consequence of aging on monocyte phenotype, the results of monocyte immunophenotyping between the young (19–30 years old, *n* = 120) and elderly (55–86 years old, *n* = 103) cohorts implied that the relative proportion of three monocyte subsets was changed by age with the percentage of classical subset (79.62 ± 6.76 *vs* 75.81 ± 7.93, *P* < 0.001) decreasing notably, the percentage of intermediate subset (4.72 ± 3.38 *vs* 5.09 ± 2.88, *P* = 0.059) rising nominally and the percentage of nonclassical subset (7.02 ± 3.43 *vs* 10.50 ± 4.82, *P* < 0.001) increasing significantly with age (Fig. 1B-D). Next, we investigated the correlation of the proportion of total monocytes and three monocyte subsets with age in the older group, indicating that only the percentage of total monocytes (*r* = 0.247, *P* = 0.012) and the intermediate subset (*r* = 0.229, *P* = 0.02) were highly associated with age (Fig. 1E-H).

### The transcriptional consequences of aging on three monocyte subsets were distinct

**Fig. 2:**
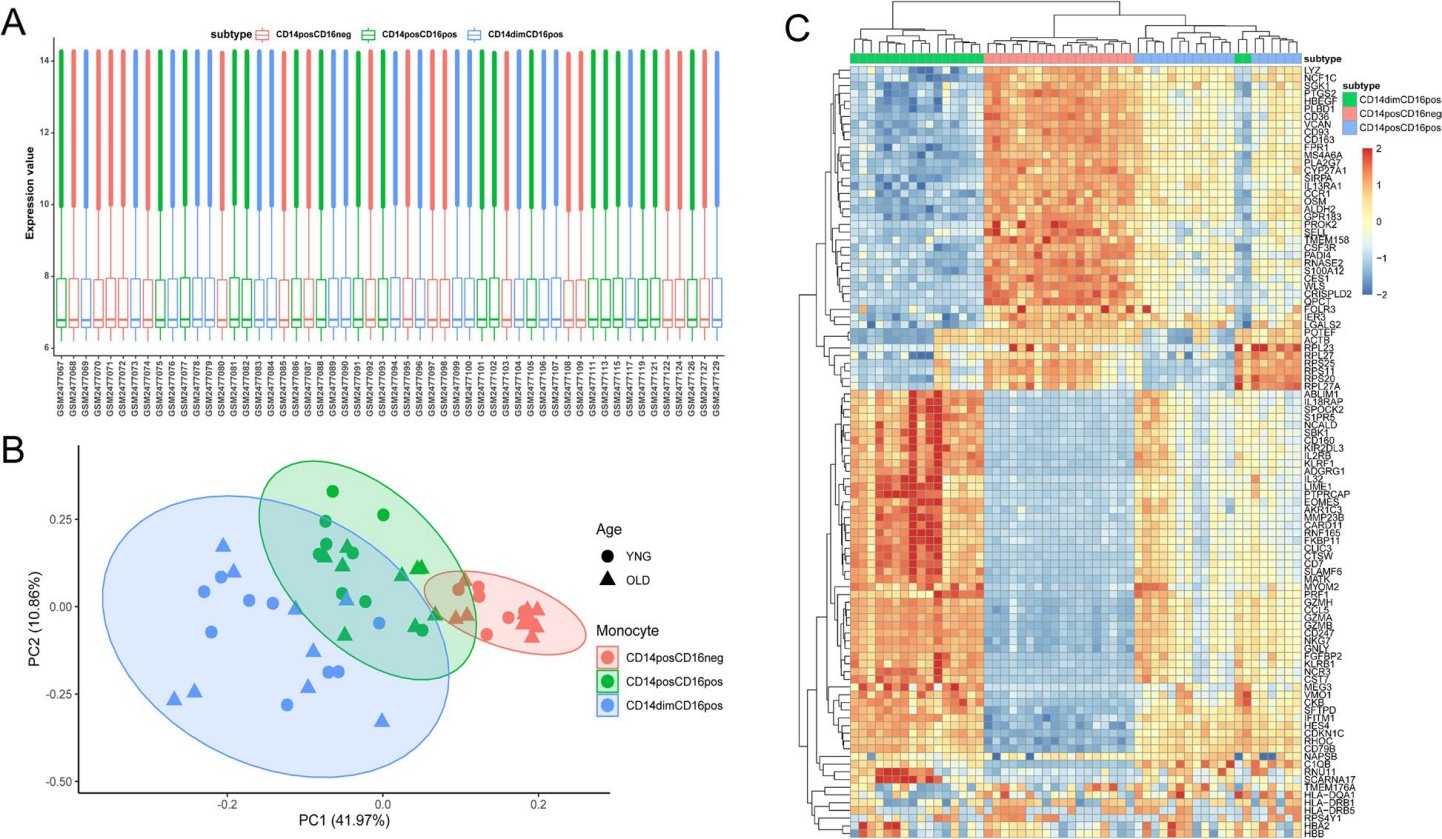
Transcriptional analysis of three monocyte subsets in young (24–36 years, *n* = 9) and aged (67–83 years, *n* = 9) individuals. **A**. Distributions of overall gene expression levels of three monocyte subsets in young and elderly individuals. Red denotes classical subset, green symbolizes intermediate subset and blue represents nonclassical subset. **B**. PCA plot of overall gene expression levels of three monocyte subsets in young and elderly individuals. Each point denotes a sample from one subset, indicated by color: classical subset (red), intermediate subset (green) and nonclassical subset (blue). Circle represents young individuals and triangle symbolizes aged persons. **C**. Heatmap of hierarchical clustering analysis of the top 100 discriminant genes independent of age for each monocyte subset: classical subset (red), intermediate subset (blue) and nonclassical subset (green). Red represents elevated gene expression and blue symbolizes decreased gene expression

**Fig. 3:**
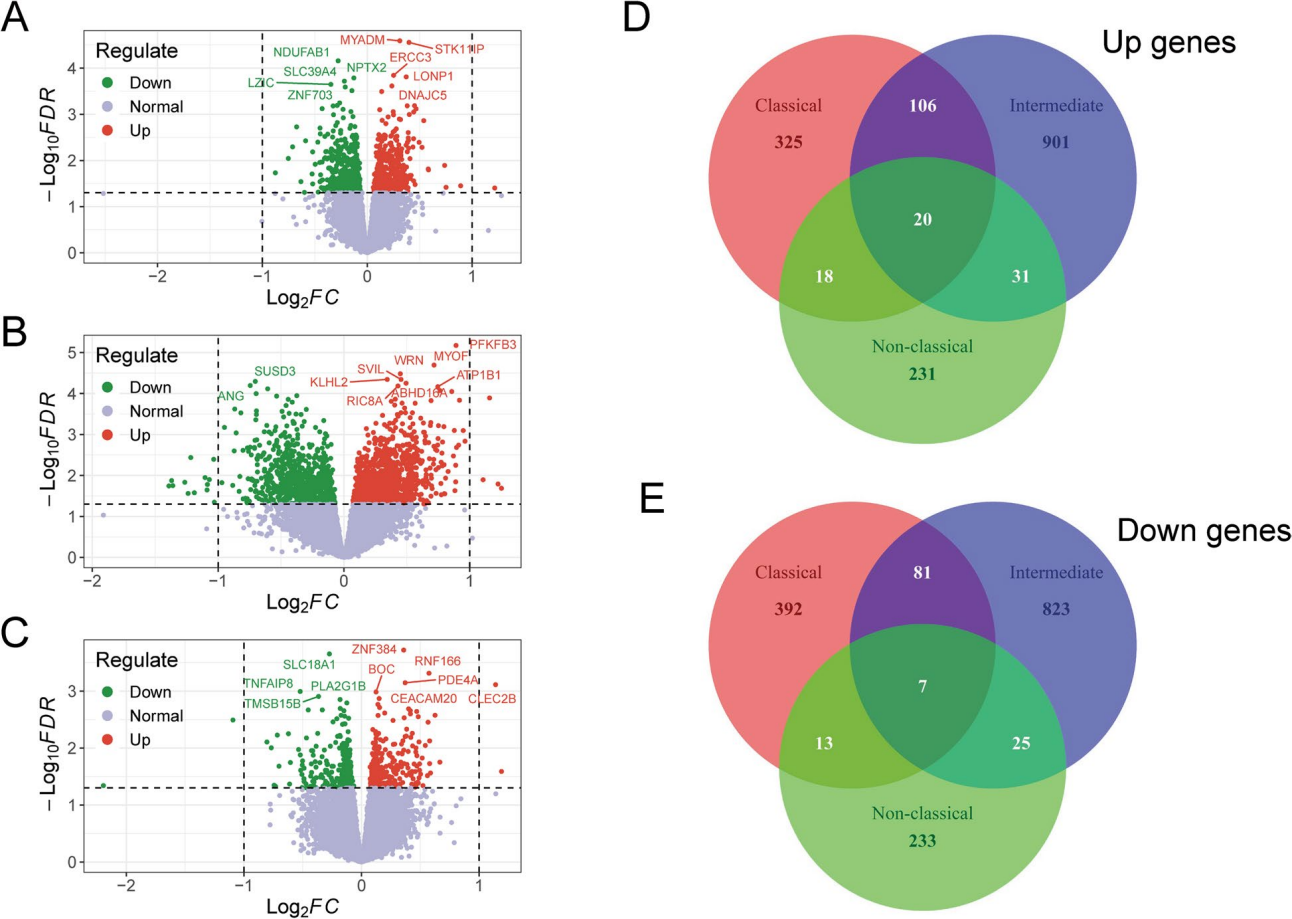
Differential expression analysis of three monocyte subsets between young (24–36 years, *n* = 9) and aged (67–83 years, *n* = 9) individuals. **A**. Volcano plot shows differential expression result of classical subset between young and old individuals. Each dot denotes a gene, red dots symbolize upregulated genes and green dots represent downregulated genes. **B**. Volcano plot presents differential expression analysis result of intermediate subset between young and aged individuals. Each dot represents a gene, red dots denote upregulated genes and green dots symbolize downregulated genes. **C**. Volcano plot depicts expression analysis result of nonclassical subset between young and elderly individuals. Each dot symbolizes a gene, red dots represent upregulated genes and green dots denote downregulated genes. **D**. Venn diagram analysis of the intersecting upregulated genes for three monocyte subsets. **E** Venn diagram analysis of the overlapping downregulated genes for three monocyte subsets

Gene expression profiles of three monocyte subsets between young and aged individuals from GSE94499 displayed good consistency (Fig. 2A). A multidimensional scaling (MDS) plot by PCA exhibited that three monocyte subsets occupied nonoverlapping spaces and there was no clear separation in the same monocyte subset between the young and older cohorts (Fig. 2B), demonstrating that three monocyte subsets were completely distinct cell populations at the transcriptional aspect and age-associated transcriptional changes of different monocyte subsets were relatively minor. Hierarchical clustering analysis of top 100 discriminant genes independent of age revealed that the intensities of gene expression for intermediate subset were in between classical and nonclassical subsets (Fig. 2C), further reinforcing a concept that the intermediate subset might represent a transient differentiation stage between classical and nonclassical subsets. Differential analysis of three monocyte subsets between young and elderly groups showed that 469 genes were upregulated and 493 genes were downregulated in the classical subset (Fig. 3A), 1058 upregulated genes and 936 downregulated genes were identified in the intermediate subset (Fig. 3B) and 300 upregulated genes and 278 downregulated genes were altered in the nonclassical subset (Fig. 3C). The majority of differential genes associated with age among three monocyte subsets were not perfectly overlapped and the intersections between classical and intermediate subsets were relatively large both in upregulated and downregulated differential genes (Fig. 3D, E), suggesting that the transcriptional effects of aging on three monocyte subsets might be distinct.

### Age-related transcriptomic analysis of CD14^+^ monocytes from 1202 individuals

**Fig. 4:**
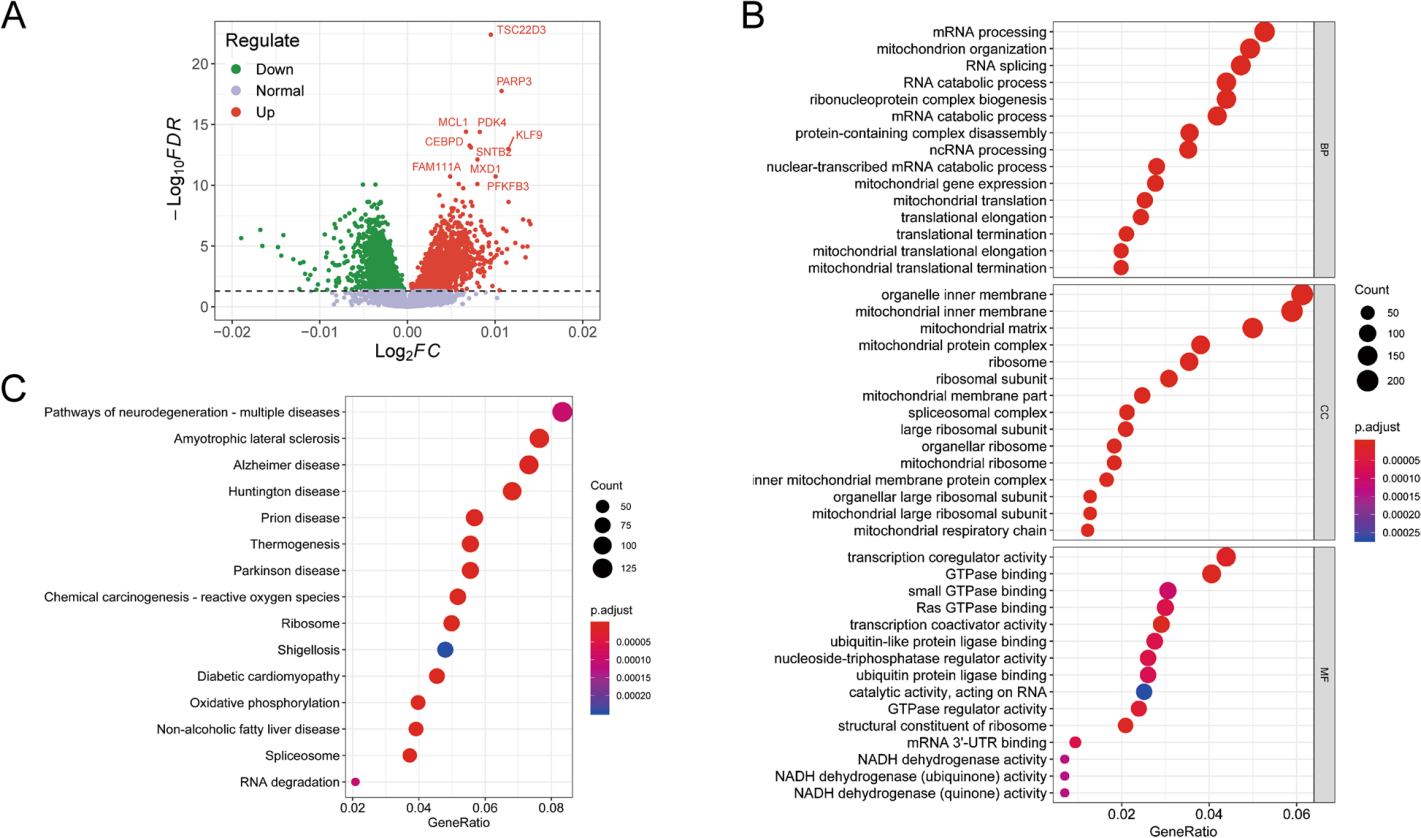
Age-related transcriptomic analysis and enrichment analyses of CD14^+^ monocytes from 1202 individuals between 44 and 83 years old. **A**. Volcano plot describes age-related differential expression result for 1202 human CD14^+^ monocyte samples. Each dot denotes a gene, red dots represent upregulated genes and green dots symbolize downregulated genes. **B**. GO function enrichment analysis of age-related differentially expressed genes, including biological process (BP), cellular component (CC) and molecular function (MF). **C**. KEGG pathway enrichment analysis of age-related differentially expressed genes

**Fig. 5:**
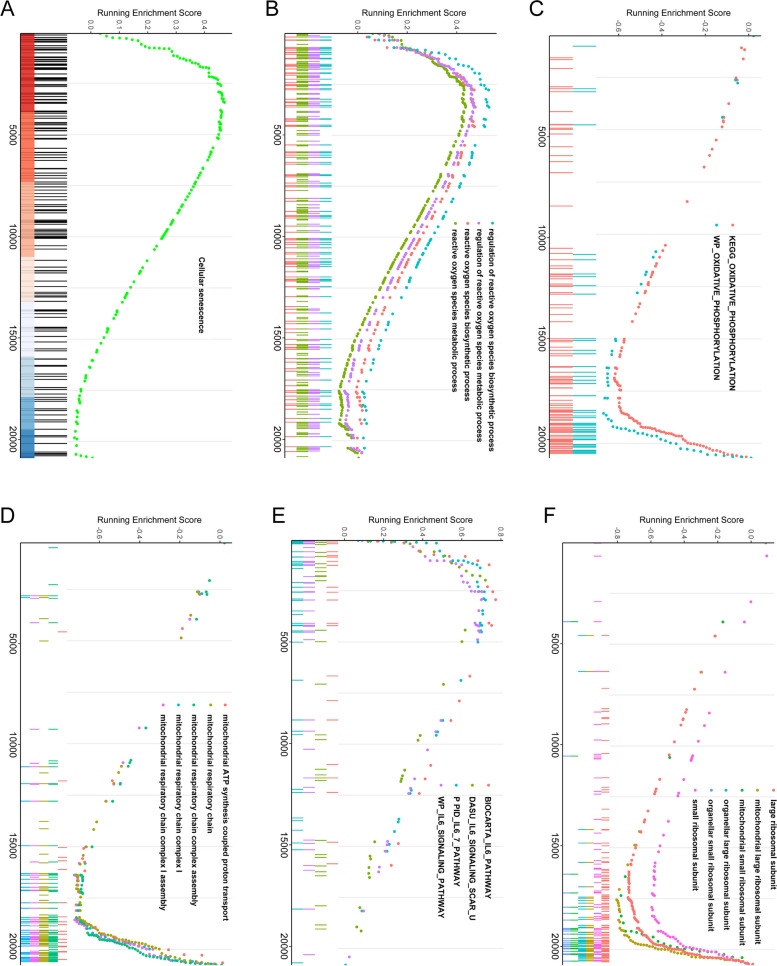
GSEA enrichment curves of age-related differential gene analysis. **A**. The increase of cellular senescence pathway in the ranked list of age-related differential genes. **B**. The upregulation of ROS pathway in the ranked list of age-associated differential genes. **C**. The downregulation of oxidative phosphorylation pathway in the ranked list of age-correlated differential genes. **D**. The decrease of mitochondrial respiratory chain pathway in the ranked list of age-related differential genes. **E**. The elevation of IL-6 pathway in the ranked list of age-associated differential genes. **F**. The decline of ribosomal structure related pathway in the ranked list of age-correlated differential genes

Given that the magnitude of age-related transcriptional differences was relatively slight and required high statistical power to be detected, we re-analysed a large publicly accessible dataset, which profiled purified CD14^+^ monocytes from 1202 individuals between the age of 44 and 83 years, and identified 2012 upregulated genes and 1827 downregulated genes accompanied with age (Fig. 4A). In addition, GO function enrichment analysis implied that age-correlated differential genes were primarily enriched in RNA splicing and processing, mitochondrial structure and respiration, ribosomal structure and function, GTPase and NADH dehydrogenase activity (Fig. 4B). Pathways associated with ROS, ribosome, spliceosome, oxidative phosphorylation and pathways related to aging diseases such as Alzheimer, Parkinson and Non-alcoholic fatty liver were identified by the KEGG pathway enrichment analysis (Fig. 4C). Furthermore, GSEA also indicated that a number of pathways were significantly altered with age, including an increase of cellular senescence, ROS and IL-6 pathways and a decrease of oxidative phosphorylation, mitochondrial respiratory chain and ribosomal structure related pathways (Fig. 5A-F).

### Increased β-galactosidase activities and ROS contents of three monocyte subsets during ageing

**Fig. 6:**
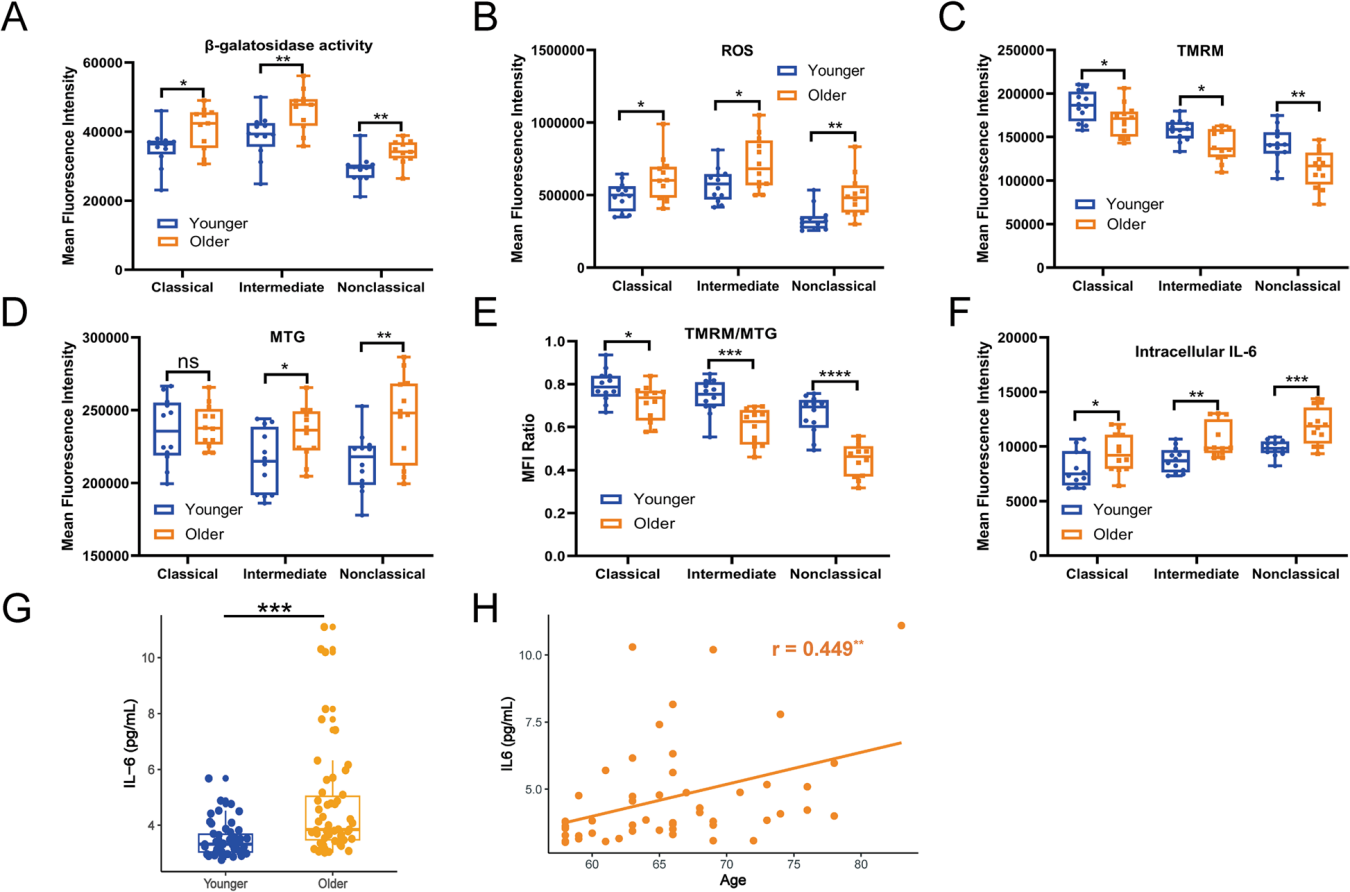
During aging, all three monocyte subsets exhibited several hallmarks of senescence and nonclassical subset was the most prominent. **A**. β-galactosidase activities of three monocyte subsets in young (21–30 years, *n* = 12) and older (60–70 years, *n* = 12) individuals. **B**. ROS contents of three monocyte subsets between young (21–30 years, *n* = 12) and elderly (60–70 years, *n* = 12) individuals. **C**. Mitochondrial contents of three monocyte subsets in young (21–30 years, *n* = 12) and old (60–70 years, *n* = 12) individuals. **D**. MMPs of three monocyte subsets between young (21–30 years, *n* = 12) and elderly (60–70 years, *n* = 12) individuals. **E**. The ratios of MMP to mitochondrial content in three monocyte subsets between young (21–30 years, *n* = 12) and aged (60–70 years, *n* = 12) individuals. **F**. The intracellular IL-6 levels of three monocyte subsets between young (21–30 years, *n* = 12) and older (60–70 years, *n* = 12) individuals. **G**. The plasma IL-6 levels between young (21–30 years, *n* = 51) and elderly (58–83 years, *n* = 50) individuals. **H**. Correlation of plasma IL-6 levels and age in the older group (58–83 years, *n* = 50). Blue dots symbolize young adults and orange dots denote aged individuals, ^*^P < 0.05, ^**^P < 0.01, ^***^P < 0.001, ^****^P < 0.0001

An increase of the β-galactosidase activity was a hallmark for the onset of senescence, we evaluated the β-galactosidase activities of three monocyte subsets in young and older cohorts using flow cytometry. As expected, monocytes from aged individuals showed significantly higher levels of β-galactosidase activities in classical (35338 ± 5452 *vs* 40927 ± 6194, *P* = 0.032), intermediate (38826 ± 6362 *vs* 46303 ± 6039, *P* = 0.009) and nonclassical (29198 ± 4116 *vs* 34048 ± 3432, *P* = 0.006) subsets, compared with young individuals (Fig. 6A).

Cell redox homeostasis was usually unbalanced with aging, showing tendency to produce oxidants and excessive ROS, which might cause oxidative damage to various macromolecules, including DNA, lipids and proteins. ROS contents of classical (493083 ± 97349 *vs* 613818 ± 158561, *P* = 0.035), intermediate (571480 ± 117055 *vs* 716967 ± 182743, *P* = 0.03) and nonclassical (335551 ± 83721 *vs* 494627 ± 145923, *P* = 0.0035) subsets from elderly individuals were all elevated, compared with those from young persons (Fig. 6B).

### Albeit MMPs of three monocyte subsets all declined with aging, intermediate and nonclassical subsets from older individuals contained more mitochondria

The high ROS contents in monocytes from aged persons, as well as the downregulation of oxidative phosphorylation and mitochondrial respiratory chain pathways, prompted us to assess mitochondrial functions between young and older cohorts. As MMP was the driving force for mitochondrial ATP synthesis and it could reflect the function of mitochondria, we measured MMP by analyzing the absorption of the membrane potential sensitive TMRM dye and quantified the total mitochondrial content by the fluorescence intensity of MTG in three monocyte subsets from young and elderly individuals. We observed that MMP levels of classical (186088 ± 17704 *vs* 169346 ± 18626, *P* = 0.034), intermediate (157682 ± 12526 *vs* 140145 ± 18062, *P* = 0.011) and nonclassical (140575 ± 20875 *vs* 113712 ± 21816, *P* = 0.006) monocytes were all decreased (Fig. 6C), while mitochondrial contents of intermediate (214625 ± 21589 *vs* 234271 ± 18035, *P* = 0.024) and nonclassical (214382 ± 19873 *vs* 244748 ± 29829, *P* = 0.008) monocytes increased with age (Fig. 6D). After normalized with mitochondrial content, MMP levels of classical (0.793 ± 0.075 *vs* 0.711 ± 0.084, *P* = 0.02), intermediate (0.740 ± 0.081 *vs* 0.601 ± 0.084, *P* < 0.001) and nonclassical (0.657 ± 0.088 *vs* 0.446 ± 0.078, *P* < 0.0001) subsets from aged persons were all significantly lower than young individuals (Fig. 6E).

### Increased intracellular IL-6 of three monocyte subsets and plasma IL-6 during aging

Previous pathway enrichment analysis indicated that an increase of IL-6 pathway was accompanied with CD14^+^ monocytes aging. Thus, we measured intracellular IL-6 levels in three monocyte subsets after LPS stimulation and monensin blockade. The results showed that monocytes from older groups had obviously higher IL-6 levels in classical (7990 ± 1630 *vs* 9439 ± 1769, *P* = 0.049), intermediate (8750 ± 1110 *vs* 10514 ± 1584, *P* = 0.005) and nonclassical (9833 ± 735 *vs* 11839 ± 1663, *P* < 0.001) subsets, as compared to young cohorts (Fig. 6F). Furthermore, we also measured soluble IL-6 concentration in plasma and found that IL-6 levels (3.48 ± 0.60 *vs* 4.69 ± 1.94, *P* < 0.001) were significantly increased in aged individuals (Fig. 6G). Meanwhile, significant positive correlation between IL-6 levels and age was also found in elderly individuals (*r* = 0.449, *P* = 0.001) (Fig. 6H).

### Impaired mitochondrial oxidative phosphorylation respiration and enhanced glycolysis of CD14^+^ monocytes during aging

**Fig. 7:**
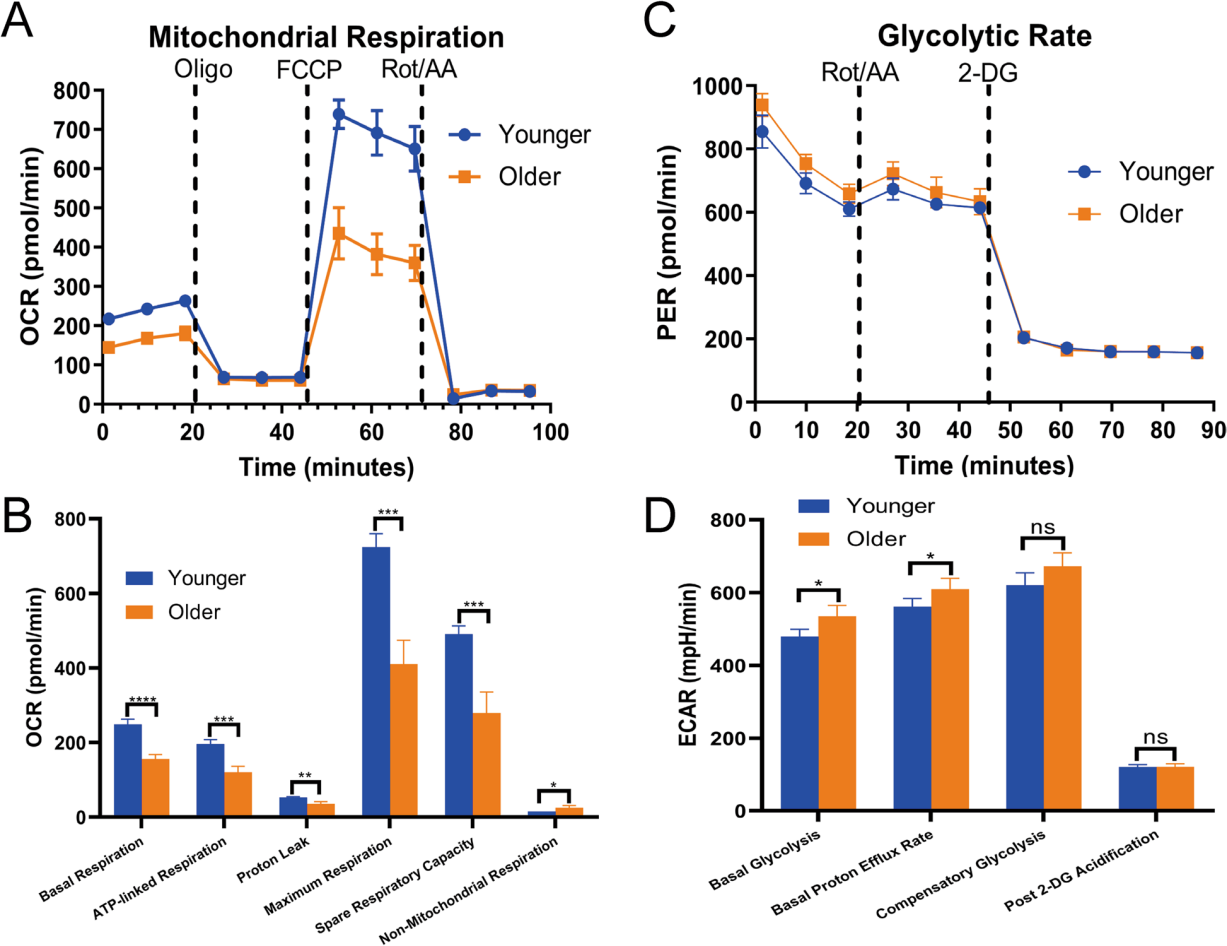
Impaired mitochondrial oxidative phosphorylation respiration and enhanced glycolysis of CD14^+^ monocytes during aging. **A**. Mitochondrial stress curves of CD14^+^ monocytes in young (24–26 years, *n* = 6) and older (62–66 years, *n* = 6) individuals. **B**. Comparisons of mitochondrial respiratory parameters of CD14^+^ monocytes between young (24–26 years, *n* = 6) and aged (62–66 years, *n* = 6) individuals. **C**. Glycolytic rate curves of CD14^+^ monocytes in young (24–26 years, *n* = 6) and old (62–66 years, *n* = 6) individuals. **D**. Comparisons of glycolytic parameters of CD14^+^ monocytes between young (24–26 years, *n* = 6) and elderly (62–66 years, *n* = 6) individuals. Blue denotes young individuals and orange represents aged individuals, ^*^P < 0.05, ^**^P < 0.01, ^***^P < 0.001, ^****^P < 0.0001

Since there is a very low proportion of intermediate and nonclassical subsets relative to classical subset, while sufficient cells were required for measurement, CD14^+^ monocytes were selected as a surrogate to assess the influence of aging on monocyte energy metabolism. Sequential injections of oligomycin, FCCP and rotenone/antimycin A were applied to calculate the parameters of oxidative phosphorylation respiration (Fig. 7A). The results suggested that basal respiration (248.81 ± 13.96 *vs* 155.93 ± 12.03, *P* < 0.0001), ATP production (195.99 ± 11.83 *vs* 120.44 ± 16.05, *P* < 0.001), proton leak (52.81 ± 2.14 *vs* 35.49 ± 5.76, *P* = 0.001), FCCP-induced maximal OCR (724.10 ± 36.19 *vs* 410.43 ± 63.97, *P* < 0.001) and spare respiratory capacity (490.37 ± 22.55 *vs* 279.43 ± 55.97, *P* < 0.001) were all notably decreased in monocytes from elderly individuals, while nonmitochondrial respiration (15.08 ± 0.12 *vs* 24.93 ± 6.22, *P* = 0.019) was significantly elevated (Fig. 7B).

Rotenone/antimycin A and 2-DG were sequentially supplemented to evaluate glycolysis levels (Fig. 7C), showing that monocytes from aged individuals had a significant increase of basal glycolysis (479.23 ± 20.08 *vs* 535.04 ± 29.89, *P* = 0.015) and basal proton efflux rate (PER) (561.30 ± 22.83 *vs* 609.33 ± 30.05, *P* = 0.034), compared with young persons (Fig. 7D).

## Discussion

In this study, we conducted a comprehensive and elaborate analysis on the influence of aging on the three monocyte subsets (CD14^+^CD16^−^, CD14^+^CD16^+^, CD14^−^CD16^+^). Consistent with previous studies [25–27], we also observed that the percentage of classical subset decreased and the proportion of nonclassical subsets increased with age. However, the percentage of intermediate subset in our study was only marginally increased, probably due to the use of different gating strategies for flow cytometry analysis and the differences in enrollment criteria of aged individuals. Transcriptome analyses of three monocyte subsets from young and aged individuals indicated that three monocyte subsets were completely dissimilar cell populations, and intermediate subset might represent a transient differentiation stage between classical and nonclassical subsets at the transcriptional aspect. In accord with this, classical monocytes were the first subset to repopulate the circulation after an acute monocytopenia, followed by intermediate subset and lastly non-classical subset, hinting that monocytes release from the bone marrow as classical monocytes, and then differentiate into intermediate and non-classical monocytes [15]. However, recent functional studies of monocyte subsets had identified that intermediate monocytes were a distinctive and highly activated monocyte subset with strengthened responsiveness to some stimulus [5, 26]. The influence of aging on the transcriptional alterations of different monocyte subsets was distinct and minor. Large sample size was required to detect this small-magnitude age-associated alterations, so we analyzed the expression profiles of CD14^+^ monocytes from 1202 individuals, and observed that age-related differential genes were significantly enriched in cellular senescence, ROS, oxidative phosphorylation, mitochondrial respiratory chain, IL-6 and ribosome-related pathways. Functional analyses of these significant pathways in three monocyte subsets between young and aged individuals verified that the β-galactosidase activities, ROS contents, intracellular IL-6 levels in three monocyte subsets and plasma IL-6 levels from elderly persons were all significantly elevated, as compared to young individuals, while MMPs were apparently decreased with age and the mitochondrial contents were only increased in intermediate and non-classical subsets. Comparing with the classical and intermediate subsets, non-classical monocytes have been previously reported to exhibit the clearest characteristics of senescence including proliferative capacity, telomere length, cellular ROS level and MMP [28]. Conversely, our study has some contradictory results, likely arising from different recruitment criteria for subject selection and experimental protocols including the use of cultured or purified monocytes, which is known to affect monocyte phenotype [29] and TLR response [30]. Consistent with the previously described that aging impaired mitochondrial respiration in classical monocytes [31], CD14^+^ monocytes from older persons had obviously lower basal and sparse respiratory capacity and significantly higher basal glycolysis than those from young individuals. In contrast with classical and intermediate subsets, the increased degree of β-galactosidase activities, ROS contents and intracellular IL-6 levels and the decreased degree of MMPs in nonclassical subsets from aged individuals were all most significant. We speculated that the mitochondrial respiratory function of nonclassical subset from older adults was the most severely impaired, requiring more glycolysis to meet the demand for energy metabolism.

During aging, the related pathways of mitochondrial electron transport chain components were apparently downregulated, which were obviously associated with age-related diseases [32, 33]. In concordance with this, we detected higher ROS contents and lower MMPs in three monocyte subsets from aged individuals. ROS could be originated from injured mitochondrial, or due to elevated NADPH oxidase activity caused by proinflammatory signals. Nonetheless, chronic exposure to ROS can also contribute to mitohormesis, which acts as a defense mechanism against mitochondrial oxidants [34–36]. The proinflammatory cytokines such as IL-6, TNF-α and IL-1β increase gradually with age, leading to a state of persistent, chronic and low-grade inflammation, called inflammaging [37, 38]. Monocytes from aged individuals have been shown to exhibit a weakened pro-inflammatory response to TLR1/2 agonists [25], our results indicated that the intracellular IL-6 levels of three monocyte subsets from elderly adults were both significantly elevated than those from young individuals following LPS stimulation, further reinforcing the notion that aging has a different effect on individual TLR responses in monocytes. However, definite evidences suggested that vascular smooth muscle cells secreted more IL-6 with aging [39, 40], the specific cellular origins of increased IL-6 in plasma are required to be further identified. IL-6 is a multifunctional cytokine that regulates mitochondrial function in various cells including increasing mitochondrial calcium levels in CD4^+^ T cells [41], reducing MMP in islets [42] and decreasing oxygen consumption in skeletal muscle [43]. A recent aging study in mice implied that aged mice had reduced OCR and enhanced mitophagy in the aortas compared with young mice, accompanied by a rise in TLR9, MYD88 and IL-6 levels [40]. Intriguingly, blocking IL-6 of elderly mouse aortas in vitro could heighten the OCR and decrease Parkin levels [40]. This study also identified a positive feedback loop between mitochondrial dysfunction and increased IL-6 levels within the aorta during aging, which could accelerate atherosclerosis [40]. Therefore, targeting inhibition of IL-6 pathway could improve bioenergetics of aging vessels and mitigate vascular aging.

The detrimental influences of aging on energy metabolism and protein synthesis are main features of aging [44]. The aging process in multiple organisms is companied with ribosome biogenesis hypofunction, including an overall reduction of protein synthesis and the age-associated decrease of rRNA expression, showing that both RNA polymerase II- and III-dependent transcription are disturbed [45–47]. In accord with this, ribosome related pathways of monocyte were also significantly downregulated with aging. The latest reports suggested that proteins misfolding occurred frequently during early ribosome biogenesis and misloaded proteins often stuck to each other, which not only inactivated its normal function, but also formed toxic aggregates [48]. To avoid this serious consequence, there was a specialized ribosome-associated quality control (ROC) mechanism existed to degrade and remove the misfolded defective proteins [49, 50]. During aging, the function of ribosome deteriorated, and the frequency of ribosome pauses and collisions notably increased, which aggravated stagnation and misfolding in protein synthesis, contributing to ROC overload and dysfunction, ultimately resulting in nascent polypeptides aggregation and proteostasis debacle [48]. To satisfy increased energy demands, many immune cells upregulated glycolytic pathway for ATP generation during their activation [51, 52]. The downregulated pathway of oxidative phosphorylation, reduced MMP, decreased spare respiratory capacity and enhanced basal glycolysis in aged monocyte hinted that the energy metabolism has switched from oxidative phosphorylation to aerobic glycolysis with age. This metabolic programming is related to “trained innate immunity”, which might augment inherent responses against pathogens, probably leading to worsen inflammatory outcome [53, 54]. Monocytes from elderly individuals have some resemblances to “trained” innate cells but also have some apparent discrepancies. The shift toward aerobic glycolysis in normal monocytes and macrophages is conducive to attain quick energy supply and produce metabolites to execute the instantaneous defensive function, comprising cytokine secretion, oxidative burst for bactericidal function, phagocytosis and antigen presentation for acquired immunity [55–57]. However, these functions in monocytes from older adults are impaired on account of the cellular energy shortage [18, 31]. The metabolic fitness of monocytes from aged individuals is possibly injured with reduced mitochondrial reserved respiratory capacity and restricted additional glucose utilization, eventually contributing to energy deficit for ribosomal biogenesis [36, 58].

Some possible limitations of our study should be acknowledged. First, we only compare the differences of monocytes and their subsets between young and aged donors, which cannot directly reflect the characteristic alterations of monocytes during normal aging. Second, owing to methodological limitations, the mitochondrial stress and glycolytic rate of each monocyte subset are not assessed, more sensitive and accurate detection techniques in the future are necessitated. Finally, this study only focuses on the aging changes of monocytes under conventional flow cytometry immunophenotyping, while mass cytometry and single-cell transcriptomics have enabled further subtyping of these monocytes.

## Conclusions

In summary, monocytes exhibited senescence-associated secretory phenotype, mitochondrial dysfunction, decreased oxidative phosphorylation respiration and increased glycolysis during aging and the nonclassical subset displayed the clearest features of aging. Our study comprehensively analyzed age-related transcriptional alterations of three monocyte subsets and identified the pivotal pathways of monocyte senescence, which may have significant implications for tactics to alleviate age-related conditions.

## Materials and methods

### Study population

Healthy young donors between 19 and 30 years old (*n* = 120) and healthy elderly donors between 55 and 86 years old (*n* = 103) were enrolled in the study. Information regarding lifestyle, clinical history and medication usage was collected from each participant by a screening questionnaire. Individuals with any previous history of infection (hepatitis B, hepatitis C or HIV), inflammatory diseases (dermatitis, colitis, Crohn’s disease, rheumatoid arthritis or lupus) or cancer were excluded. Smokers and individuals with regular use of anti-inflammatory or cholesterol-lowering medications were also excluded. 2 mL whole blood was collected from each young (*n* = 120) or older (*n* = 103) individual for hematological measurement, monocyte immunophenotyping and the following functional validation of three monocyte subsets during aging. This study was approved by the Ethics Committees of Zhongnan Hospital of Wuhan University (2020195) and all individuals gave their written informed consent prior to enter the study.

### Hematological measurement and monocyte immunophenotyping

Routine blood indicators including red blood cell (RBC) count, hematocrit (HCT), mean corpuscular volume (MCV), red blood cell distribution width coefficient variation (RDW-CV), hemoglobin (HGB), mean corpuscular hemoglobin (MCH), mean corpuscular hemoglobin concentration (MCHC), white blood cell (WBC) count, neutrophil (NEUT) count, percentage of neutrophils (NEUT%), lymphocyte (LYMPH) count, percentage of lymphocytes (LYMPH%), monocyte (MONO) count, percentage of monocytes (MONO%), eosinophil (EO) count, percentage of eosinophils (EO%), basophil (BASO) count, percentage of basophils (BASO%), platelet (PLT) count and mean platelet volume (MPV) were determined using a Beckman Coulter DxH 800 automated blood analyzer (Beckman, USA) within 30 min of blood draw. For each donor, 100 μL whole blood was labeled with a mixture of three mouse anti-human monoclonal fluorochrome-conjugated antibodies (10 μL anti-CD86-APC (BD Biosciences, USA), 10 μL anti-CD14-FITC (BD Biosciences, USA) and 3 μL anti-CD16-BV421 (BD Biosciences, USA)) and incubated at 4 °C in the dark for 30 min. Subsequently, red blood cells were lysed by 1 × Red Blood Cell Lysis Buffer (Solarbio, Beijing, China) at 4 °C in the dark for 15 min and then washed twice with PBS. Stained cells were analyzed using Beckman CytoFLEX S flow cytometer (Beckman Coulter, CA, USA) and Beckman CytExpert software version 2.4 (Beckman Coulter, CA, USA). Monocytes (P1) were first gated in FSC/SSC dot plot (Fig. S1A), followed by CD86/SSC dot plot, identifying CD86^+^ monocytes (P2) (Fig. S1B). Finally, CD86^+^ monocytes were classified into classical (CD14^+^CD16^−^), intermediate (CD14^+^CD16^+^) and non-classical (CD14^−^CD16^+^) subsets based on the CD14/CD16 dot plot (Fig. S1C).

### Transcriptional analyses of three monocyte subsets between young and aged individuals

GSE94499 (https://www.ncbi.nlm.nih.gov/geo/query/acc.cgi?acc=GSE94499) was downloaded from Gene Expression Omnibus (GEO) database, which consisted of gene expression profiles of three purified monocyte subsets from 9 young donors between 24 and 36 years old and 9 aged non-frail donors between 67 and 83 years old. Principal component analysis (PCA) was utilized to assess transcriptional differences between three monocyte subsets and the impacts of aging on gene expression. Differential expression analyses of three monocyte subsets between young and elderly individuals were performed individually by limma package and the significance threshold was set to an unadjusted *P* < 0.05.

### Transcriptomic analysis of CD14^+^ monocytes from 1202 individuals between 44 and 83 years old

GSE56045 (https://www.ncbi.nlm.nih.gov/geo/query/acc.cgi?acc=GSE56045) was acquired from GEO database, which comprised transcriptomic profiles of purified CD14^+^ monocytes from 1202 individuals ranging from 44 to 83 years of age in the Multi-Ethnic Study of Atherosclerosis (MESA) cohort. Differential gene expression was conducted by a linear model using limma package and we accounted for confounding variables by incorporating race-gender-site and chip parameters into multiple linear regression model: gene ~ age + race-gender-site + chip. Age was used as continuous quantitative variable, no partition into age groups was conducted. *P* values were adjusted for multiple testing correction by the Benjamini–Hochberg method and the significance threshold was set to false discovery rate (*FDR*) < 0.05. Differential genes were then subjected to Gene Ontology (GO) and Kyoto Encyclopedia of Genes and Genomes (KEGG) pathway enrichment analyses in clusterProfiler package. Considering that some transcriptional changes were not obvious but still had important biological significance, gene set enrichment analysis (GSEA) via the fgsea package was applied to identify significantly altered pathways and draw enrichment curves.

### Detection of β-galactosidase activities in three monocyte subsets

CellEvent™ senescence green flow cytometry assay kit (Thermo Fischer Scientific, USA) was used to determine β-galactosidase activities of three monocyte subsets. For each donor, 300 μL whole blood was lysed and stained with a mixture of 20 μL anti-CD86-APC (BD Biosciences, USA), 20 μL anti-CD14-PE (BD Biosciences, USA) and 5 μL anti-CD16-BV421 (BD Biosciences, USA) at 4 °C for 30 min and fixed in 4% paraformaldehyde for 10 min at room temperature, then incubated with the 1 × CellEvent™ Senescence Green Probe for 90 min in a 37 °C incubator without CO_2_. Fluorescence was measured at maximum wavelengths of 490 nm (excitation)/514 nm (emission).

### Measurement of reactive oxygen species contents in three monocyte subsets

For each donor, 300 μL whole blood was lysed and incubated with 10 μM DCFH-DA (UE Landy, China) for 30 min at 37 °C, and then labeled with 20 μL anti-CD86-APC (BD Biosciences, USA), 20 μL anti-CD14-PE (BD Biosciences, USA) and 5 μL anti-CD16-BV421 (BD Biosciences, USA) at 4 °C for 30 min. The maximum excitation and emission wavelengths of the fluorescent products were 504 nm and 529 nm.

### Determination of mitochondrial contents and mitochondrial membrane potentials in three monocyte subsets

For each donor, 600 μL whole blood was divided into two equal parts for measurement of mitochondrial contents and mitochondrial membrane potentials (MMPs), respectively. 300 uL whole blood was lysed and incubated with 100 nM MitoScene™ Green I (UE Landy, China) for 30 min at 37 °C, followed by staining with 20 μL anti-CD86-APC (BD Biosciences, USA), 20 μL anti-CD14-PE (BD Biosciences, USA) and 5 μL anti-CD16-BV421 (BD Biosciences, USA) at 4 °C for 30 min. The fluorescent probe for mitochondrial content was detected at maximum excitation and emission wavelengths in 490 nm and 523 nm.

Another 300 μL whole blood was lysed and incubated with 100 nM TMRM (UE Landy, China) for 30 min at 37 °C, and then labelled with 20 μL anti-CD86-APC (BD Biosciences, USA), 20 μL anti-CD14-FITC (BD Biosciences, USA) and 5 μL anti-CD16-BV421 (BD Biosciences, USA) at 4 °C for 30 min. The fluorescent dye for MMP was detected at maximum wavelengths of 548 nm (excitation)/ 573 nm (emission).

### Measurement of intracellular IL-6 levels in three monocyte subsets and determination of IL-6 levels in plasma

For each donor,1 mL whole blood was diluted 1:1 with RPMI-1640 culture medium and stimulated with a final concentration of 100 ng/mL LPS (Sigma-Aldrich, St Louis, MO, USA) for 2 h in a humidified 37 °C incubator. To inhibit protein secretion and accumulate cytokines within monocytes, Monensin (Beyotime, China) was supplemented for another 4 h at a final concentration of 2 μM. After red blood cells were lysed, the cell suspension was stained with 20 μL anti-CD86-APC (BD Biosciences, USA), 20 μL anti-CD14-FITC (BD Biosciences, USA) and 5 μL anti-CD16-BV421 (BD Biosciences, USA) at 4 °C for 30 min. Cells were then fixed and permeabilized with fixation/permeabilization buffer (BD Biosciences, USA) during 20 min at 4 °C. After washing, rat anti-human IL-6-PE was added and incubated for 30 min at 4 °C. Cells were washed and resuspended in FACS buffer until analysis on a Beckman CytoFLEX S flow cytometer (Beckman Coulter, CA, USA). Plasma IL-6 level was measured on Roche Cobase 801 Electrochemiluminescence Analyzer (Roche, Switzerland) according to the manufacturer’s instruction.

### Seahorse mitochondrial stress and glycolytic rate assays of CD14^+^ monocytes

Peripheral blood mononuclear cells (PBMCs) were isolated by density gradient centrifugation in Ficoll Plus 1.077 g/cm^3^ (Solarbio, Beijing, China) and washed twice with PBS. Subsequently, monocytes were purified from PBMCs with anti-human CD14 monoclonal antibody-coated magnetic microbeads (Miltenyi) using magnetic-activated cell sorted (MACS) system (Miltenyi, Bergisch Gladbach, Germany) in accordance with the manufacturer’s protocol. CD14^+^ monocytes were spun down onto plates coated with poly-D-lysine (Solarbio, Beijing, China) at 1.0 × 10^6^ cells per well. Measurements of oxygen consumption rates (OCR) and extracellular acidification rates (ECAR) based on fluorescent sensors were conducted on a Seahorse XFe24 extracellular flux analyzer (Agilent, Santa Clara, CA, USA) consistent with the manufacturer’s recommendations. For mitochondrial stress test, sequential injections of 1.5 μM oligomycin (ATP synthase inhibitor), 2 μM carbonyl cyanide 4-(trifluoromethoxy) phenylhydrazone (FCCP) (mitochondrial uncoupler) and 0.5 μM rotenone/antimycin A (complex I and III inhibitors) were added to the assay medium to assess mitochondrial respiratory function. Spare respiratory capacity was calculated as maximal OCR values minus basal OCR values. For glycolytic rate assay, 0.5 μM rotenone/antimycin A and 50 mM 2-Deoxy-D-glucose (2-DG) (hexokinase inhibitor) were injected subsequently into assay medium to evaluate glycolytic function. Basal glycolysis was computed as basal ECAR values subtracted the contribution of CO_2_ to extracellular acidification derived from mitochondrial respiration.

### Statistical analysis

Qualitative indicators were presented as frequencies with percentages and the Chi-square was utilized to compare the differences between two groups. Continuous parameters were expressed as mean with standard deviation (SD) and normality distribution was evaluated by the Kolmogorov–Smirnov test. Independent *t*-test and Mann–Whitney *U*-test were applied to examine the differences between two groups with or without normal distribution, respectively. In the correlation analysis of two variables, we used Pearson correlation test when the assumptions satisfied a normal distribution. Otherwise, the spearman correlation test was applied. All data analyses were conducted by R software (version 3.6.0) and a threshold of two-sided *P* < 0.05 was considered to be statistically significant.

### Supplementary Information

**Additional file 1:** **Figure S1.** Flow gating strategy for distinguishing three monocyte subsets. A. Monocytes (P1) were first gated in FSC/SSC dot plot. B. CD86^+^ monocytes (P2) were identified in CD86/SSC dot plot. C. CD86^+^ monocytes were classified into classical (CD14^+^CD16^-^), intermediate (CD14^+^CD16^+^) and non-classical (CD14^-^CD16^+^) subsets based on the CD14/CD16 dot plot. **Figure S2.** Detection of β-galactosidase activities in three monocyte subsets between young and aged individuals. A. The negative control of the β-gal assay. B. The positive control of the β-gal assay. C. The representative histogram of β-galactosidase activity in classical subset from young individual. D. The representative histogram of β-galactosidase activity in classical subset from aged individual. E. The representative histogram of β-galactosidase activity in intermediate subset from young individual. F. The representative histogram of β-galactosidase activity in intermediate subset from aged individual. G. The representative histogram of β-galactosidase activity in non-classical subset from young individual. H. The representative histogram of β-galactosidase activity in non-classical subset from aged individual. **Figure S3.** Measurement of reactive oxygen species (ROS) contents in three monocyte subsets between young and aged individuals. A. The negative control of ROS content. B. The positive control of ROS content. C. The representative histogram of ROS content in classical subset from young individual. D. The representative histogram of ROS content in classical subset from aged individual. E. The representative histogram of ROS content in intermediate subset from young individual. F. The representative histogram of ROS content in intermediate subset from aged individual. G. The representative histogram of ROS content in non-classical subset from young individual. H. The representative histogram of ROS content in non-classical subset from aged individual. **Figure S4.** Determination of mitochondrial contents in three monocyte subsets between young and aged individuals. A. The negative control of mitochondrial content. B. The positive control of mitochondrial content. C. The representative histogram of mitochondrial content in classical subset from young individual. D. The representative histogram of mitochondrial content in classical subset from aged individual. E. The representative histogram of mitochondrial content in intermediate subset from young individual. F. The representative histogram of mitochondrial content in intermediate subset from aged individual. G. The representative histogram of mitochondrial content in non-classical subset from young individual. H. The representative histogram of mitochondrial content in non-classical subset from aged individual. **Figure S5.** Detection of mitochondrial membrane potentials (MMPs) in three monocyte subsets between young and aged individuals. A. The negative control of MMP. B. The positive control of MMP. C. The representative histogram of MMP in classical subset from young individual. D. The representative histogram of MMP in classical subset from aged individual. E. The representative histogram of MMP in intermediate subset from young individual. F. The representative histogram of MMP in intermediate subset from aged individual. G. The representative histogram of MMP in non-classical subset from young individual. H. The representative histogram of MMP in non-classical subset from aged individual.

## Data Availability

The datasets generated and/or analyzed during the current study are available in the GEO repository, GSE94499 (https://www.ncbi.nlm.nih.gov/geo/query/acc.cgi?acc=GSE94499) and GSE56045 (https://www.ncbi.nlm.nih.gov/geo/query/acc.cgi?acc=GSE56045).

## Abbreviations

MMP: Mitochondrial membrane potential
ROS: Reactive oxygen species
LPS: Lipopolysaccharide
SASP: Senescence-associated secretory phenotype
RBC: Red blood cell count
HCT: Hematocrit
MCV: Mean corpuscular volume
RDW-CV: Red blood cell distribution width coefficient variation
HGB: Hemoglobin
MCH: Mean corpuscular hemoglobin
MCHC: Mean corpuscular hemoglobin concentration
WBC: White blood cell count
NEUT: Neutrophil count
NEUT%: Percentage of neutrophils
LYMPH: Lymphocyte count
LYMPH%: Percentage of lymphocytes
MONO: Monocyte count
MONO%: Percentage of monocytes
EO: Eosinophil count
EO%: Percentage of eosinophils
BASO: Basophil count
BASO%: Percentage of basophils
PLT: Platelet count
MPV: Mean platelet volume
GEO: Gene Expression Omnibus
PCA: Principal component analysis
MESA: Multi-Ethnic Study of Atherosclerosis
FDR: False discovery rate
GO: Gene Ontology
KEGG: Kyoto Encyclopedia of Genes and Genomes
GSEA: Gene set enrichment analysis
PBMCs: Peripheral blood mononuclear cells
MACS: Magnetic-activated cell sorted
OCR: Oxygen consumption rates
ECAR: Extracellular acidification rates
FCCP: Carbonyl cyanide 4-(trifluoromethoxy) phenylhydrazone
2-DG: 2-Deoxy-D-glucose
2-NBDG: 2-(N-(7-Nitrobenz-2-oxa-1,3-diazol-4-yl)Amino)-2-Deoxyglucose
MDS: Multidimensional scaling
ROC: Ribosome-associated quality control
