# Supplementary material for: Transcriptional characteristics and functional validation of three monocyte subsets during aging
Source: Immun Ageing. 2023 Sep 27;20:50. doi: 10.1186/s12979-023-00377-1 (PMC10523626; doi:10.1186/s12979-023-00377-1)
Supplement: Supplementary file 1 — Additional file 1: Figure S1. Flow gating strategy for distinguishing three monocyte subsets. A. Monocytes (P1) were first gated in FSC/SSC dot plot. B. CD86+ monocytes (P2) were identified in CD86/SSC dot plot. C. CD86+ monocytes were classified into classical (CD14+CD16-), intermediate (CD14+CD16+) and non-classical (CD14-CD16+) subsets based on the CD14/CD16 dot plot. Figure S2. Detection of β-galactosidase activities in three monocyte subsets between young and aged individuals. A. The negative control of the β-gal assay. B. The positive control of the β-gal assay. C. The representative histogram of β-galactosidase activity in classical subset from young individual. D. The representative histogram of β-galactosidase activity in classical subset from aged individual. E. The representative histogram of β-galactosidase activity in intermediate subset from young individual. F. The representative histogram of β-galactosidase activity in intermediate subset from aged individual. G. The representative histogram of β-galactosidase activity in non-classical subset from young individual. H. The representative histogram of β-galactosidase activity in non-classical subset from aged individual. Figure S3. Measurement of reactive oxygen species (ROS) contents in three monocyte subsets between young and aged individuals. A. The negative control of ROS content. B. The positive control of ROS content. C. The representative histogram of ROS content in classical subset from young individual. D. The representative histogram of ROS content in classical subset from aged individual. E. The representative histogram of ROS content in intermediate subset from young individual. F. The representative histogram of ROS content in intermediate subset from aged individual. G. The representative histogram of ROS content in non-classical subset from young individual. H. The representative histogram of ROS content in non-classical subset from aged individual. Figure S4. Determination of mitochondr [file 12979_2023_377_MOESM1_ESM.docx]

**Supplementary material:**


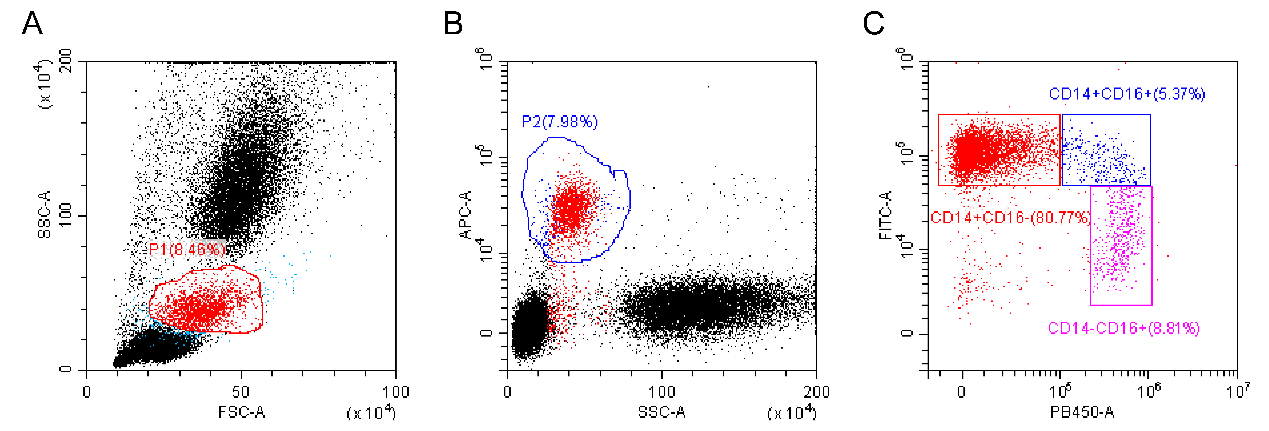


**Fig. S1** Flow gating strategy for distinguishing three monocyte subsets. **A**. Monocytes (P1) were first gated in FSC/SSC dot plot. **B**. CD86^+^ monocytes (P2) were identified in CD86/SSC dot plot. **C**. CD86^+^ monocytes were classified into classical (CD14^+^CD16^-^), intermediate (CD14^+^CD16^+^) and non-classical (CD14^-^CD16^+^) subsets based on the CD14/CD16 dot plot.


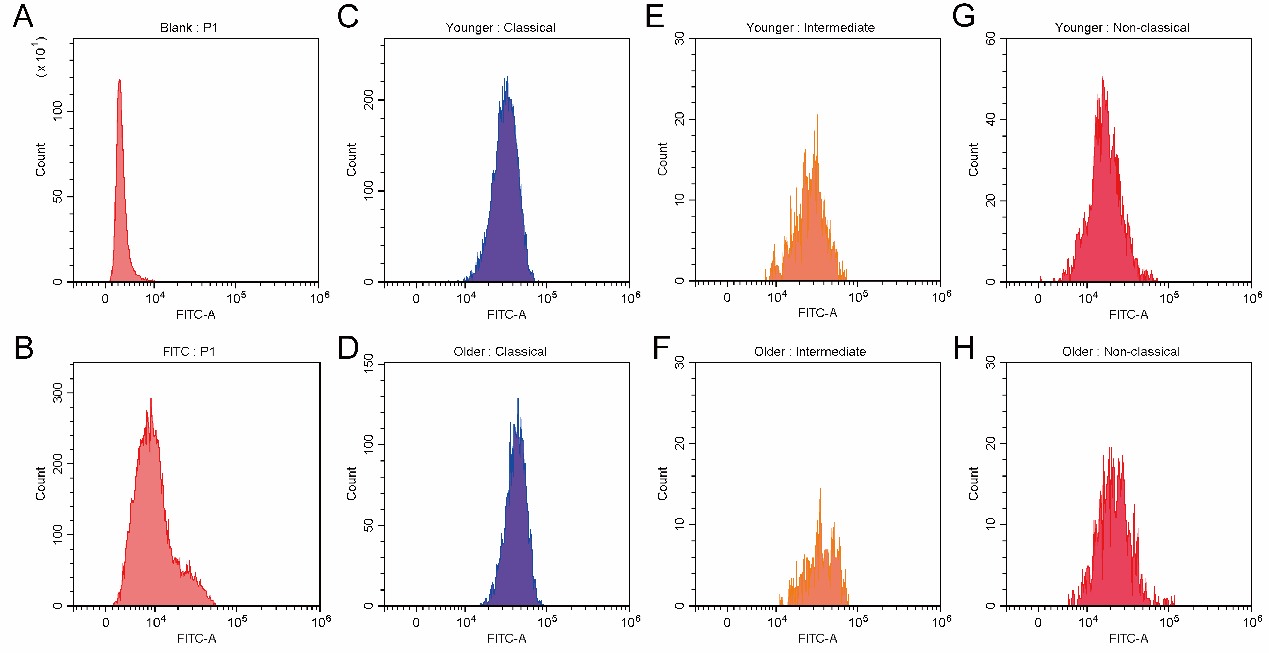


**Fig. S2** Detection of β-galactosidase activity in three monocyte subsets between young and aged individuals. **A**. The negative control of the β-gal assay. **B**. The positive control of the β-gal assay. **C**. The representative histogram of β-galactosidase activity in classical subset from young individual. **D**. The representative histogram of β-galactosidase activity in classical subset from aged individual. **E**. The representative histogram of β-galactosidase activity in intermediate subset from young individual. **F**. The representative histogram of β-galactosidase activity in intermediate subset from aged individual. **G**. The representative histogram of β-galactosidase activity in non-classical subset from young individual. **H**. The representative histogram of β-galactosidase activity in non-classical subset from aged individual.


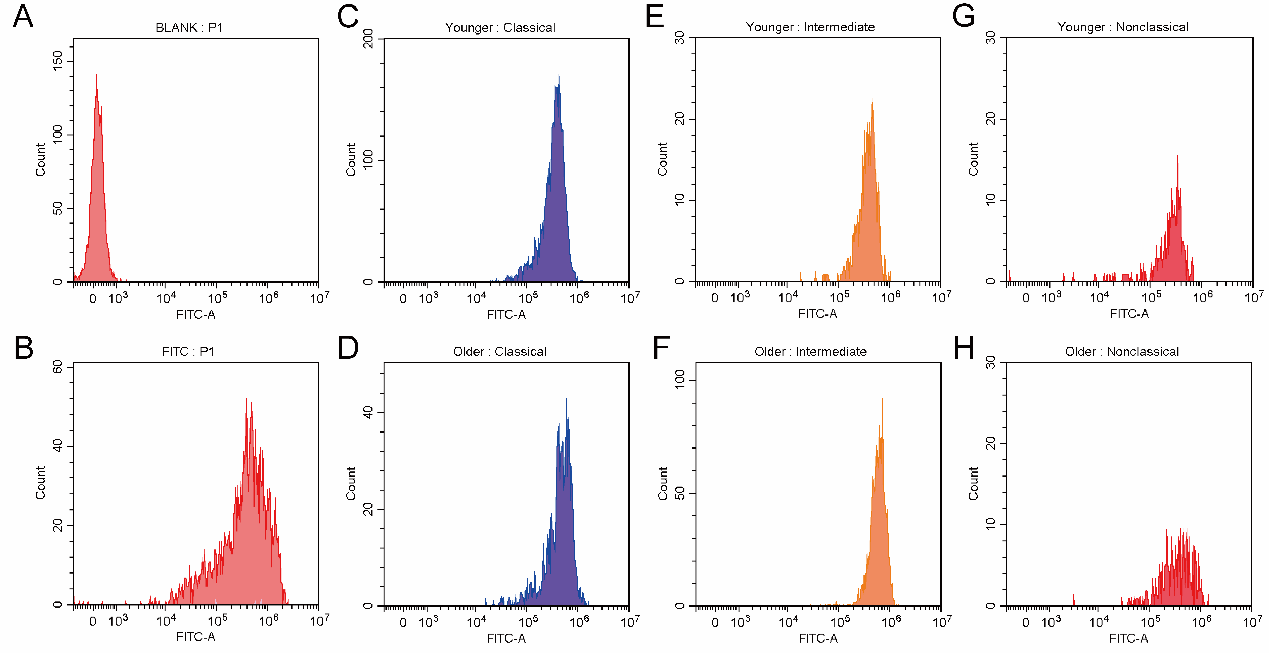


**Fig. S3** Measurement of reactive oxygen species (ROS) content in three monocyte subsets between young and aged individuals. **A**. The negative control of ROS content. **B**. The positive control of ROS content. **C**. The representative histogram of ROS content in classical subset from young individual. **D**. The representative histogram of ROS content in classical subset from aged individual. **E**. The representative histogram of ROS content in intermediate subset from young individual. **F**. The representative histogram of ROS content in intermediate subset from aged individual. **G**. The representative histogram of ROS content in non-classical subset from young individual. **H**. The representative histogram of ROS content in non-classical subset from aged individual.


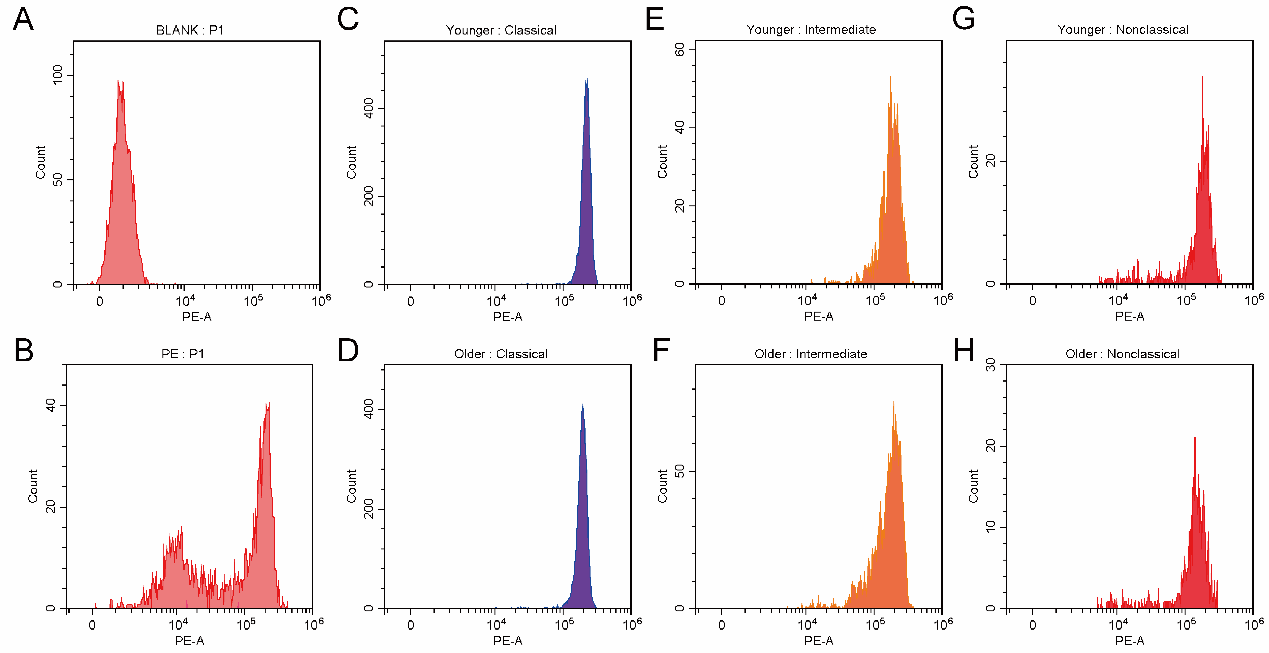

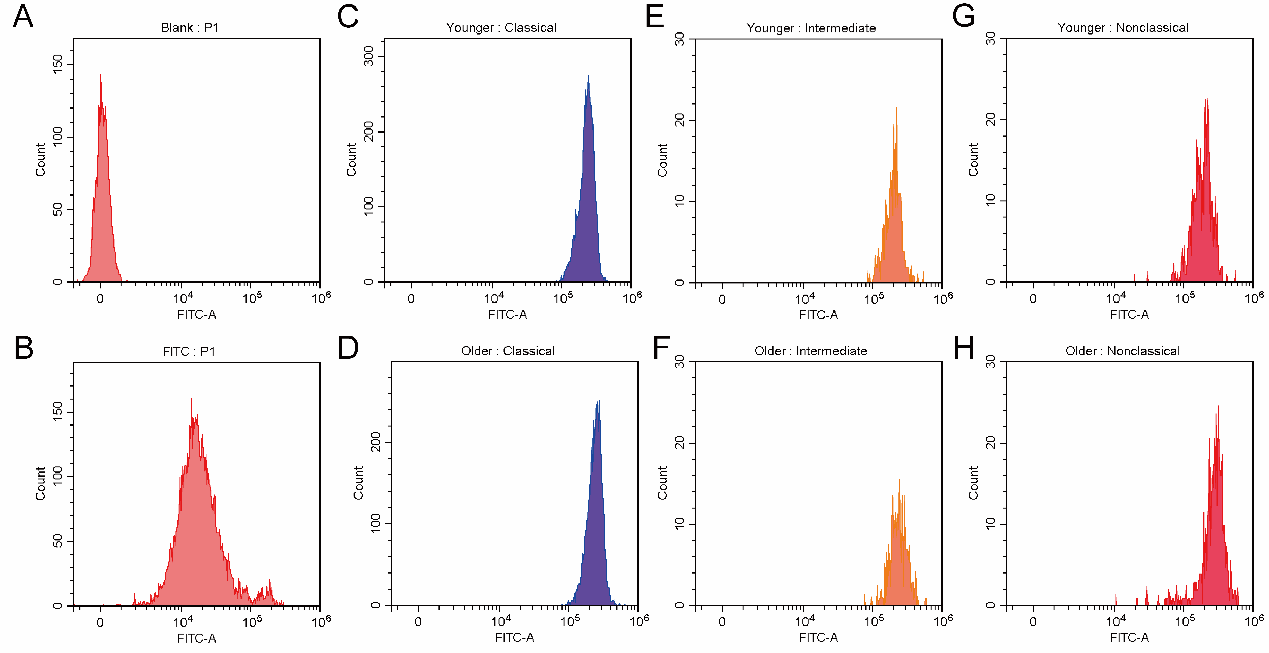
**Fig. S4** Determination of mitochondrial content in three monocyte subsets between young and aged individuals. **A**. The negative control of mitochondrial content. **B**. The positive control of mitochondrial content. **C**. The representative histogram of mitochondrial content in classical subset from young individual. **D**. The representative histogram of mitochondrial content in classical subset from aged individual. **E**. The representative histogram of mitochondrial content in intermediate subset from young individual. **F**. The representative histogram of mitochondrial content in intermediate subset from aged individual. **G**. The representative histogram of mitochondrial content in non-classical subset from young individual. **H**. The representative histogram of mitochondrial content in non-classical subset from aged individual.

**Fig. S5** Detection of mitochondrial membrane potential (MMP) in three monocyte subsets between young and aged individuals. **A**. The negative control of MMP. **B**. The positive control of MMP. **C**. The representative histogram of MMP in classical subset from young individual. **D**. The representative histogram of MMP in classical subset from aged individual. **E**. The representative histogram of MMP in intermediate subset from young individual. **F**. The representative histogram of MMP in intermediate subset from aged individual. **G**. The representative histogram of MMP in non-classical subset from young individual. **H**. The representative histogram of MMP in non-classical subset from aged individual.
